# In vivo optical quality of posterior-chamber phakic implantable collamer lenses with a central port

**DOI:** 10.1186/s40662-021-00251-5

**Published:** 2021-08-16

**Authors:** Robert Montés-Micó, Francisco Pastor-Pascual, Enrique Artiaga-Elordi, Ramón Ruiz-Mesa, Pedro Tañá-Rivero

**Affiliations:** 1 Optics and Optometry & Vision Sciences Department, University of Valencia, c/o Dr Moliner 50, 46100 Valencia, Spain; 2 Oftalvist Clinic, Valencia, Spain

**Keywords:** Implantable collamer lens, Phakic, Optical quality, Higher order aberrations, Modulation transfer function

## Abstract

**Background:**

The aim of this review is to summarize the optical quality results in patients following the implantation of the V4c implantable collamer lens with a central port (ICL, STAAR Surgical Inc.).

**Main text:**

A literature search in several databases was carried out to identify those publications, both prospective, retrospective and/or comparative with other refractive surgery procedures, reporting optical outcomes of patients who were implanted with the V4c ICL model. A total of 17 clinical studies published between 2012 and 2021 were included in this review. A detailed analysis of the available data was performed including number of eyes, follow-up and preoperative spherical equivalent. Specifically, the review focused on several optical parameters including higher-order aberrations (HOAs), modulation transfer function (MTF) cut-off frequency and Strehl ratio. This review encompassed a total of 817 eyes measured using different optical devices based on Hartmann-Shack, retinal image quality measurement and ray-tracing technologies at different follow-ups.

**Conclusions:**

The outcomes found in this review lead us to conclude that the ICL V4c model provides good optical quality, by means of different metrics, when implanted.

## Background

The use of phakic intraocular lenses for the correction of different degrees of refractive error has expanded in the last decades. Different designs and models have been shown as safe and predictable for correcting myopia, hyperopia and astigmatism. The outcomes of posterior-chamber, iris-fixated anterior-chamber and angle-supported anterior-chamber intraocular lenses have been compiled in different review publications [1–4]. These also focused on the selection criteria, possible complications and differences in designs available in the market.

One of the most used phakic intraocular lens type worldwide is the implantable collamer lens (ICL; STAAR Surgical Inc., Monrovia, USA). The most recent model of this lens appeared in the market 10 years ago; the V4c ICL, which incorporates a 0.36-mm central hole in the center, named KS Aquaport, to avoid the need for iridectomies or iridotomies. This model has been extensively used in the last decade and different clinical studies from 2012 to date have shown that its implantation is a safe and efficient procedure with low rates of adverse events. Several review articles published at different times supported this conclusion and discussed clinical studies with short-, mid- and long-term follow-ups [5–7]. These publications, in addition to reporting possible complications, focused mainly on the visual and refractive outcomes as well as on other parameters including intraocular pressure, endothelial cell density or vault measurements. However, to date no review studies reporting and discussing the in vivo optical quality that this lens provides to our patients have been published. Considering that having a good optical quality is mandatory in order to provide good visual quality, it then becomes necessary to carry out a complete and updated analysis of the in vivo optical quality outcomes reported in the peer-review literature with this lens.

Thus, the purpose of the present manuscript is to provide an updated review of the in vivo optical quality outcomes that the phakic V4c myopic ICL model provides when implanted, in the context of clinical studies published in peer-reviewed journals.

## Main text

### Methods

Three databases were explored to search for clinical studies reporting outcomes with the ICL V4c model: PubMed (U.S. National Library of Medicine), Web of Science (Clarivate Analytics) and Scopus (Elsevier). Only publications in peer-reviewed journals written in English were considered. These publications, retrospective, prospective and/or comparative with other refractive surgery procedures, were analyzed in detail looking for data on optical quality (studies reporting outcomes in eyes with ocular pathologies were not considered). The date of the last electronic search was March 14, 2021. The search included a combination of the following keywords: “implantable collamer lens”,” ICL”,” phakic lens”,” V4c’,” optical quality “and” aberrations”. In addition, for each selected publication, all of its references were also assessed to ensure that any relevant publication on this topic would not be missed.

Different information was extracted from each publication, including: authors, year of publication, title of the study, journal of publication, sample size (number of eyes), follow-up time (maximum), preoperative spherical equivalent (SE), higher-order aberrations (HOAs), pupil size for HOAs measurement, modulation transfer function (MTF) cut-off frequency, Strehl ratio and device used to measure the optical quality. Mean, standard deviation and ranges were included for all parameters analyzed, wherever available.

### Results

A total of 17 articles, published between 2012 and 2021, resulted from the literature search following the criteria indicated. Table 1 shows a summary of these 17 clinical studies reporting optical quality outcomes of eyes implanted with the V4c ICL [8–24]. Authors, number of eyes included, follow-up, preoperative SE and instruments used to measure optical quality are shown. Wan et al. [17] had the largest sample of all 17 studies with 137 eyes, and Baptista et al. [22] had the longest follow-up period (38.5 months). Average preoperative SE across all the studies was − 8.90 D, but for individual SE value it ranged from − 4.26 D [21] to − 14.01 D [17]. The 817 eyes in these studies were measured using different optical devices based on Hartmann-Shack (*n* = 530 eyes), retinal image quality measurement (*n* = 357) and ray-tracing technologies (*n* = 48). Hartmann-Shack instruments used were the KR-9000 (Topcon, Tokyo, Japan), VISX Wavescan (Johnson & Johnson Vision, Jacksonville, USA), KR-1 W (Topcon, Tokyo, Japan) and WASCA (Carl Zeiss Meditec AG, Jena, Germany). The OQAS (Keeler, Malvern, USA) is based on retinal image quality measurement and the iTrace (Tracey Technologies, Houston, USA) on ray-tracing technology.

**Table. 1:** Studies reporting optical quality outcomes of eyes implanted with the ICL V4c model using different devices

Authors	Year	Eyes (patients)	Follow-up	SE (D)	Device
Shimizu et al. [8]	2012	29 (29)	3 mo	−7.52 ± 2.02	KR-9000(Topcon, Tokyo, Japan)
Hosny and Shalaby [9]	2013	20 (NR)	1.5 mo	−12.9 ± 2.38	ViSX Wavescan(Johnson & Johnson Vision, Jacksonville, USA)
Kamiya et al. [10]	2014	28 (28)	3 mo	−7.17 ± 1.98	OQAS (Keeler, Malvern, USA)
Huseynova et al. [11]	2014	44 (44)	3 mo	−10.05 ± 2.72	KR-1 W (Topcon, Tokyo, Japan)
Tian et al. [12]	2017	18 (18)	1 mo	−11.44 ± 5.18	iTrace (Tracey Technologies, Houston, USA)
Park et al. [13]^a^	2017	46 (28)	3 mo	−8.58 ± 2.08	KR-1 W (Topcon, Tokyo, Japan)OQAS (Keeler, Malvern, USA)
Miao et al. [14]	2018	67 (38)	3 mo	−12.44 ± 3.15	OQAS (Keeler, Malvern, USA)
Liu et al. [15]	2018	40 (22)	3 mo	−6.12 ± 2.19	ViSX Wavescan(Johnson & Johnson Vision, Jacksonville, USA)OQAS (Keeler, Malvern, USA)
Qin et al. [16]	2019	30 (15)	3 mo	−13.87 ± 2.16	iTrace (Tracey Technologies, Houston, USA)
Wan et al. [17]^b^	2020	27 (27)29 (29)54 (54)27 (27)	6 mo	−5.33 ± 0.83−8.01 ± 0.82−10.67 ± 0.94− 14.01 ± 1.47	WASCA (Carl Zeiss Meditec AG, Jena, Germany)
Niu et al. [18]	2020	39 (20)	12 mo	−7.54 ± 1.07	OQAS (Keeler, Malvern, USA)
Wei et al. [19]	2020	94 (57)	6 mo	−8.07 ± 1.03	WASCA (Carl Zeiss Meditec AG, Jena, Germany)
Wei et al. [20]^c^	2020	42 (21)46 (23)	6 mo	−8.26 ± 1.43− 8.30 ± 1.43	WASCA (Carl Zeiss Meditec AG, Jena, Germany)
Zhang et al. [21]	2020	16 (8)	6 mo	−4.26 ± 1.55	OQAS (Keeler, Malvern, USA)
Baptista et al. [22]	2020	30 (15)	38.5 mo	−8.25 ± 2.6	OQAS (Keeler, Malvern, USA)
Chen et al. [23]	2021	59 (59)	1 week	−15.16 ± 4.77	OQAS (Keeler, Malvern, USA)
Aruma et al. [24]	2021	32 (20)	12 mo	−5.21 ± 0.73	WASCA (Carl Zeiss Meditec AG, Jena, Germany)OQAS (Keeler, Malvern, USA)

*SE* spherical equivalent; *D* diopters; *NR* not reported; *mo* months

^a^: data on centered lenses; ^b^: data of 4 subgroups with SE ≤ 6.0 D, SE from −6.13 to −9.0 D, SE from − 9.13 to −12.0 D and SE from − 12.13 to 18.0 D (from top to bottom); ^c^: data of 2 subgroups of non-toric (top) and toric lenses (bottom)

Table 2 describes in detail the values found for HOAs at different pupils for the different studies using Hartmann-Shack and ray-tracing instruments when data is available. Table 3 summarizes the mean and standard deviation MTF cut-off frequency and Strehl ratio values found in those studies using the OQAS instrument (4.0-mm pupil). Note that three studies used more than one instrument providing both values for HOAs and MTF cut-off frequency and Strehl ratio [13, 15, 24].

**Table. 2:** Studies reporting higher-order aberrations (microns) using Hartmann-Shack or ray-tracing devices at different pupil diameters

Authors	HOAs(4.0-mm)	HOAs(5.0-mm)	HOAs(6.0-mm)	HOAs(^a^-mm)	Device
Shimizu et al. [8]	0.12 ± NR	–	0.38 ± NR	–	KR-9000 (Topcon, Tokyo, Japan)
Hosny and Shalaby [9]	–	–	–	0.31	ViSX Wavescan (Johnson & Johnson Vision, Jacksonville, USA)
Huseynova et al. [11]	0.29 ± NR	–	0.44 ± NR	–	KR-1 W (Topcon, Tokyo, Japan)
Tian et al. [12]	–	–	–	0.81	iTrace (Tracey Technologies,Houston, USA)
Park et al. [13]	0.13 ± 0.05	–	–	–	KR-1 W (Topcon, Tokyo, Japan)
Liu et al. [15]	0.11 ± 0.03	–	0.39 ± 0.10	–	ViSX Wavescan (Johnson & Johnson Vision, Jacksonville, USA)
Qin et al. [16]	–	0.47 ± 0.00	–	–	iTrace (Tracey Technologies,Houston, USA)
Wan et al. [17]^b^	–	–	0.38 ± 0.140.42 ± 0.180.36 ± 0.160.32 ± 0.08	–	WASCA (Carl Zeiss Meditec AG, Jena, Germany)
Wei et al. [19]	–	0.26 ± 0.07	–	–	WASCA (Carl Zeiss Meditec AG, Jena, Germany)
Wei et al. [20]^c^	–	0.27 ± 0.080.27 ± 0.12	–	–	WASCA (Carl Zeiss Meditec AG, Jena, Germany)
Aruma et al. [24]	–	0.38 ± 0.18	–	–	WASCA (Carl Zeiss Meditec AG, Jena, Germany)

*HOA* higher-order aberrations; *NR* not reported;

^a^: pupil diameter not indicated; ^b^: data of 4 subgroups with SE ≤ 6.0 D, SE from −6.13 to −9.0 D, SE from − 9.13 to −12.0 D and SE from − 12.13 to 18.0 D (from top to bottom); ^c^: data of 2 subgroups of non-toric (top) and toric lenses (bottom)

**Table. 3:** Studies reporting the modulation transfer function (MTF) cut-off frequency and Strehl ratio using the OQAS device

Authors	MTF cut-offfrequency (cpd)	Strehl ratio
Kamiya et al. [10]	26.21 ± 8.32	0.16 ± 0.04
Park et al. [13]	33.42 ± 9.20	0.19 ± 0.06
Miao et al. [14]	37.64 ± 9.74	0.21 ± 0.06
Liu et al. [15]	26.39 ± 12.08	0.15 ± 0.06
Niu et al. [18]	40.65 ± 7.31	NR
Zhang et al. [21]	31.29 ± NR	0.18 ± NR
Baptista et al. [22]	26.91 ± 9.2	NR
Chen et al. [23]^a^	37.00 ± 7.9637.89 ± 10.80	0.21 ± 0.050.20 ± 0.06
Aruma et al. [24]	36.60 ± 9.39	0.20 ± 0.06

*cpd* cycles per degree; *NR* not reported;

^a^: baseline values with (top) and without brimonidine (bottom)

## Discussion

It has been demonstrated in different clinical studies that V4c ICL implantation performed well with regards to safety, efficacy, predictability and, in those with long follow-up, stability for the correction of low, moderate and high myopia [5–7]. In addition to standard parameters for a refractive surgery procedure, some studies [8–24] assessed the optical quality of eyes implanted with these lenses in vivo to understand the optical performance they provide, specially analyzing the role of the central hole in the optical quality of the lens. In vitro optical bench studies have shown that the optical quality of the V4c ICL model is good (fulfils the International Organization for Standardization criteria for MTF) [25] and comparable to the same model without the central hole [26], and when simulated, both models provided comparable visual performance [27]. The present review aimed to discuss these important outcomes which are inherently related to the visual quality expected in our patients. Considering that the clinical studies included in this review measured the optical performance of the lens using different devices, the present discussion will be split in different sections according to the metrics reported, and finally, a comparative section with corneal refractive procedures will be included.

### Higher-order aberrations (HOAs)

Shimizu et al. [8] were the first authors reporting both clinical and optical outcomes in eyes implanted with the V4c ICL model. These authors used a Hartmann-Shack wavefront aberrometer (KR-9000) to measure the optical quality in 29 eyes 3 months after surgery and compared it with a non-hole ICL eyes (V4c ICL model was implanted in one eye and non-hole ICL in the other eye of the same patient by randomization assignment). Specifically, they reported HOAs (including coma- and spherical-like aberrations) for 4.0- and 6.0-mm pupils (see Table 2). The mean SE was − 7.52 ± 2.02 D, ranging from − 4.00 to − 11.75 D. The mean inductions of HOAs were 0.02 ± 0.05 μm and 0.08 ± 0.10 μm, for 4.0- and 6.0-mm pupils, respectively. They concluded that the V4c ICL was essentially equivalent to the non-hole ICL model with regards to the induction of HOAs. Hosny et al. [9] evaluated at 6 weeks the in vivo aberrometric performance of 20 eyes with a mean SE of − 12.9 ± 2.38 D (ranging from − 9 to − 20 D) using another Hartmann-Shack wavefront aberrometer (ViSX Wavescan). They compared the outcomes found with the anterior-chamber AcrySof Cachet intraocular lens (Alcon Labs, Fort Wort, USA). No pupil diameter was indicated for the HOAs measurement, and therefore no comparison with other studies with the V4c ICL model was possible, the authors noted that there were no statistically significant differences in the induction of HOAs between both models postoperatively (both induced small amount of negative spherical aberration that did not affect the HOAs).

Huseynova et al. [11] reported data on a large sample (*n* = 44 eyes) and compared it to that found in another group of eyes implanted with the non-hole ICL model (*n* = 21 eyes). They measured HOAs at 4.0- and 6.0-mm pupils with the KR-1 W Hartmann-Shack wavefront aberrometer (see Table 2). Note that HOAs values reported were slightly higher than those found by Simizu et al. [8], with the mean induction of HOAs also being larger: 0.15 μm and 0.73 μm, for 4.0- and 6.0-mm pupils, respectively [11]. We consider that differences in preoperative SE may play a role: − 7.52 ± 2.02 D [8] vs − 10.05 ± 2.72 D [11]. Huseynova et al. [11] concluded that none of the HOAs values showed any significant difference between hole and non-hole ICL groups. Tian et al. [12] assessed a small sample of eyes (*n* = 18) at 1-month postsurgery using the iTrace aberrometer. They reported values of HOAs but unfortunately, no pupil diameter was indicated in order to compare with previous studies. They compared them with standard non-hole ICL and concluded that HOAs were higher (about 4-times) in the V4c model (0.81 μm vs 0.22 μm, *P* < 0.05). This study disagrees with previous studies [8, 9, 11], but, as previously indicated, no direct comparison and discussion can be done due to the lack of information about the pupil.

Park et al. [13] used the KR-1 W aberrometer to measure the optical quality of eyes implanted with the V4c and the non-hole model at different degrees of decentering. Table 2 shows data on centered lenses for a 4.0-mm pupil in 46 eyes with a mean preoperative SE of − 8.58 ± 2.08 D. Mean HOAs value was similar to that found by Shimizu et al. [8]. These authors indicated that HOAs were significantly higher in eyes with a decentered central hole within one-hole diameter to two-hole diameter distance compared to eyes with the central hole within one-hole diameter from the pupil center (*P* = 0.02). They compared these with eyes implanted with the non-hole ICL model and concluded that V4c ICL implantation provided satisfactory visual quality equivalent to that provided by conventional ICL regardless of the presence of central hole and degree of decentration. This was supported by the in vitro study of Perez-Vives et al. [26] who, despite finding an increase in coma aberration with decentration of this model, concluded that this increase (comparable between both designs) was clinically negligible and did not have a significant effect on the retinal simulated images. Furthermore, in a separate study, they found that ICL decentration did not have any effect on the visual performance when simulated using adaptive optics [27].

Liu et al. [15] using the ViSX Wavescan measured HOAs in 19 eyes for a 4.0-mm pupil and 16 eyes for a 6.0-mm pupil, all implanted with the V4c ICL. HOAs values reported were similar to those found by Shimizu et al. [8] and Huseynova et al. [11] (see Table 2). They indicated that none of HOAs changed significantly after the surgery for both pupil sizes, except some trefoil, tetrafoil and 2nd astigmatism for a 6.0-mm pupil. They suggested that the increased HOAs at 6.0-mm pupil may be probably due to the relatively small optical zone of the lens (optical diameter of 5.77 mm for a − 6.00 D lens power). An in vitro study comparing ICL models with small and large optical diameters showed comparable optical quality [28] and hence, the source might be related more to the ICL surgery. Qin et al. [16] evaluated the optical quality in 30 high myopic eyes (> − 10 D) using the iTrace device (SE: − 13.87 ± 2.16 D) for a 5-mm pupil. They found a significant increase of HOAs at 1 week (0.51 μm) but decreased at 3 months postsurgery (0.47 μm), similar to those found preoperatively (0.48 μm).

Wan et al. [17] reported data on a large sample analyzing the outcomes for four degrees of myopia at 6 months postsurgery: SE ≤ 6.0 D, SE from − 6.13 to − 9.0 D, SE from − 9.13 to − 12.0 D and SE from − 12.13 to 18.0 D. They used the WASCA aberrometer reporting HOAs values for a 6.0-mm pupil. They showed that there was no significant change in the total HOAs between preoperative and postoperative values for each group. However, quadrafoil increased significantly in all groups (*P* < 0.05), trefoil also did (*P* < 0.05) except for the low degree of myopia group, and spherical aberration for eyes > − 9.0 D (*P* < 0.05). Corneal incision and tilt of the ICL [26] may be related to the increasing non-rotational symmetrical aberrations (i.e., coma) and the change in spherical aberration (negative induction) may be related to the negative spherical aberration of the own lens that increases when the ICL power increases [29]. Moreover, an increase on coma is expected since a displacement of a lens with spherical aberration generates coma [30].

Wei et al. [19] measured the optical quality in 94 eyes using the WASCA aberrometer at 6 months postsurgery. These authors reported a significant increase of HOAs after the surgery (*P* < 0.05) indicating that this increase might be caused by the procedure itself or the innate optical properties of the lenses. This agrees with that indicated by Wan et al. [17] (size and orientation of the corneal incision) and Pérez-Vives et al. [26] (tilt and decentration). Specifically, Wei et al. [19] reported a mean value of 0.26 ± 0.07 μm for a 5.0-mm pupil, which was about half to that reported by Qin et al. [16]. However, it must be pointed out that the mean SE in the study of Qin et al. [16] was larger (about − 14 D) than that reported by Wei et al. [19] (about − 8 D). As previously indicated, higher ICL power generates larger values of spherical aberration [29]. In another study, Wei et al. [20] examined with the same aberrometer 42 and 46 eyes implanted with the non-toric and toric V4c ICL model at 6 months, respectively. SE was similar between both groups of eyes and the HOAs reported at 5.0-mm pupil were also similar (see Table 2). These values were similar to those found by the same author [19] (increase of HOAs and trefoil, *P* < 0.05) and slightly lower than those reported by Qin et al. [16], as previously explained. They concluded that both toric and non-toric ICL models induced similar acceptable level of HOAs.

Recently, Aruma et al. [24] assessed 32 eyes at 1-year postsurgery using the WASCA aberrometer showing a significant increase in HOAS and vertical coma after the surgery. They found a mean HOAs value of 0.38 ± 0.18 μm for a 5.0-mm pupil. This value is smaller than that found by Qin et al. [16]. This difference may be attributed to the fact that the patients of Aruma et al. [24] had lower preoperative SE than Qin et al. [16]. However, in contrast, Wei et al. [19, 24] showed higher preoperative SE but lower HOAs than Aruma et al. [24]. Other sources, perhaps related to the surgery, might play a role (i.e., incision, tilt and/or decentration of the ICL).

### Modular transfer function (MTF) cut-off frequency and Strehl ratio

Kamiya et al. [10] analyzed 28 eyes of 28 patients implanted with the V4c ICL model at 3 months postsurgery using the OQAS instrument for a 4.0-mm pupil (SE: − 7.17 ± 1.98 D). They specifically studied both the MTF cut-off frequency (frequency at which the MTF reaches a value of 0.01: frequency up to which the eye can focus an object on the retina with a significant 1% contrast) and the Strehl ratio (ratio of the central maximum of the illuminance of the point spread function in the aberrated-eye to the central maximum that would be found in a corresponding aberration-free system) and obtained the following values: 26.21 ± 8.32 cpd (95% confidence interval, 9.91 to 42.52 cpd) and 0.16 ± 0.04 (95% confidence interval, 0.08 to 0.25), respectively (see Table 3). These authors also measured these parameters in a 24 age-matched eyes of 24 patients implanted with the non-hole ICL. They concluded that there were no significant differences in the MTF cut-off frequency (*P* = 0.59) and Strehl ratio (*P* = 0.82) between both groups of eyes, therefore both lenses provided excellent optical quality, and thus suggested that the presence of the central hole did not affect the optical quality after the surgery significantly.

Park et al. [13] measured 46 eyes (centered lenses with a mean SE of − 8.58 ± 2.08 D) with the same instrument at the same follow-up, reporting similar outcomes: 33.42 ± 9.20 cpd and 0.19 ± 0.06. They compared these values with those found in 49 eyes implanted with the non-hole ICL and showed that they were not significantly different between both groups. Miao et al. [14] found slightly higher values for 67 eyes with higher SE value (− 12.44 ± 3.15 D) at the same follow-up (see Table 3). In contrast, lower values were reported by Liu et al. [15] for 40 eyes measured at the same follow-up period (SE: − 6.12 ± 2.19 D). Notwithstanding, they indicated that there were no statistically significant differences both in MTF cut-off frequency and Strehl ratio between pre- and post-surgery (*P* = 0.67 and *P* = 0.21, respectively). Niu et al. [18] showed large MTF cut-off frequency when analyzing 39 eyes with a SE of − 7.54 ± 1.07 D at 1-year postsurgery and concluded that the optical quality of eyes implanted with this lens was good.

Zhang et al. [21] evaluated a small sample (16 eyes), showing that there was a statistical difference for MTF cut-off frequency (31.29 cpd; *P* < 0.001) but not for preoperative and postoperative (6 months) Strehl ratio values (0.18; *P* = 0.130). They indicated that implantation of this lens improved the optical quality significantly, including the MTF cut-off frequency and other parameters. This agrees with Baptista et al. [22], who assessed the optical quality of eyes implanted with the ICL V4c model and the Artiflex lens (Ophtec BV, Groningen, The Netherlands). They reported, with a mean follow-up of 38.5 ± 10.1 months, a mean MTF cut-off frequency of 26.91 ± 9.2 cpd and concluded that both lenses showed excellent optical performance. Chen et al. [23], in a specific study to assess the effect of brimonidine tartrate 0.2% solution after V4c ICL implantation, showed that both MTF cut-off frequency and Strehl ratio varied as a function of the time after the use of brimonidine (note that postoperative stabilization of the cornea is not likely to be reached 1 week after surgery). The outcomes indicated in Table 3 for baseline were good. Aruma et al. [24], in 32 eyes with a 1-year of follow-up, showed similar values to the previous study with a mean MTF cut-off frequency of 36.60 ± 9.39 cpd (ranging from 12.84 to 53.45 cpd) and a Strehl ratio of 0.20 ± 0.06 (ranging from 0.097 to 0.356).

### Comparison with corneal refractive procedures

ICL implantation has been usually performed in eyes where other corneal refractive surgeries, such as photorefractive keratectomy, laser in situ keratomileusis (LASIK) or small incision lenticule extraction (SMILE), are contraindicated due to the large amount of SE. However, the correction of low amounts of SE with this lens is increasing as an alternative to these procedures. We consider that it is interesting to analyze in detail those comparative studies between the V4c ICL model and other corneal refractive procedures that include optical quality measurements.

Liu et al. [15] compared the optical quality after V4c ICL implantation and wavefront-guided LASIK with the STAR S4 IR excimer laser (Johnson & Johnson Vision, Jacksonville, USA) and the IntraLase femtosecond laser system (Johnson & Johnson Vision, Jacksonville, USA) for flap creation. They assessed a group of 40 eyes with a mean SE of − 6.12 ± 2.19 D and a group of 40 eyes with a mean SE of − 3.27 ± 25.50 D. Due to the SE differences between groups, the authors adopted a matched pair design to analyze the pre- and postoperative optical quality changes avoiding a direct comparison between procedures. Based on this assumption, they found that ICL implantation induced fewer HOAs than wavefront-guided LASIK. This is an expected outcome given that laser ablation changes the corneal surface increasing spherical aberration specifically.

Niu et al. [18] carried out a comparative study between the V4c ICL and SMILE using the VisuMax femtosecond laser system (Carl Zeiss Meditec, AG, Jena, Germany) with matched-SE groups (*P* = 0.594). 39 eyes implanted with the V4c ICL model (SE: − 7.54 ± 1.07 D) and 37 eyes submitted to SMILE (SE: − 7.39 ± 0.98 D). They found that the MTF cut-off frequency in the V4c ICL group (40.65 ± 7.31 cpd) was significantly higher than that found in the SMILE group (37.10 ± 5.99 cpd) 1-year after surgery (*P* = 0.022). They also indicated that there was no significant difference in MTF cut-off for SMILE and ICL V4c implantation between preoperative and follow-ups. They suggested that the optical system could achieve stability shortly after both procedures, remaining stable over longer periods.

Wei et al. [19] also compared both procedures in a sample of 94 eyes implanted with the V4c ICL and 103 eyes submitted to SMILE: SE of − 7.70 ± 1.01 D and − 7.39 ± 0.79 D, respectively (*P* = 0.15). They found that HOAs (5.0-mm pupil) increased significantly after 6 months of both types of surgery, and the increase was lower in the V4c ICL group than in the SMILE group (0.03 ± 0.08 μm vs 0.16 ± 0.14 μm, respectively, *P* < 0.001). As previously discussed, ICL implantation does not change the corneal profile as it occurs during SMILE due to laser ablation, increasing as a function of the amount of myopia correction. A more oblate surface produced by SMILE increased HOAs. The increase of HOA in ICL eyes might be related to the size of the corneal incision and/or tilt and decentration of the lens as discussed in the previous section.

Aruma et al. [24] carried out a similar study with lower amounts of SE in a sample of 32 eyes implanted with the V4c ICL and 35 eyes submitted to SMILE: SE of − 5.21 ± 0.73 D and − 5.07 ± 0.67 D, respectively (*P* = 0.15). Their results agree with those found previously comparing both procedures, and found that for a 5.0-mm pupil, the changes in coma after ICL implantation were significantly less than after SMILE (*P* < 0.01). Authors suggested that this might be related to the corneal incision and healing. This study also included values from the OQAS device and showed a MTF cut-off frequency of 36.05 ± 9.76 cpd (ranging from 16.99 to 52.62 cpd) in the SMILE group, which was significantly lower than that in the ICL group: 36.60 ± 9.39 cpd (ranging from 12.84 to 53.45 cpd, *P* = 0.001). Authors discussed that this could be due to the fact that the ICL implantation guaranteed the integrity of the central cornea and avoided the effect of corneal wound healing. However, no significant differences were found for the Strehl ratio: 0.20 ± 0.05 (ranging from 0.096 to 0.289) and 0.20 ± 0.06 (ranging from 0.097 to 0.356) for the SMILE and V4c ICL group, respectively (*P* = 0.57).

Siedlecki et al. [31] indicated that studies of corneal refractive procedures compared to ICL implantation showed lower induction of HOAs, which agrees with previous studies comparing the optical and visual quality between the non-hole ICL model and LASIK that concluded that ICL provided better outcomes in MTF [32]. They discussed that it may be hypothesized that some shortcomings of corneal refractive surgery compared with ICL implantation in high myopia might be ameliorated by SMILE based for example on larger functional optical zone sizes and improved preservation of the cornea’s natural asphericity, thereby resulting in lower surgically induced HOAs. These authors concluded that ICL implantation induced fewer corneal HOAs. As a general remark we wish to highlight that there is a change to an oblate corneal profile after corneal refractive surgery and it remains prolate after ICL implantation, which obviously reduce the induction of HOAs. However, it should be considered that ICL implantation may induce HOAs both from the corneal incision (size and location), optics of the own lens (the higher the power, the higher the spherical aberration) and its position (centration and/or tilt) when implanted.

## Conclusions

The good optical quality outcomes reported in the different clinical studies analyzed in the present review support the use of the V4c ICL model. Different studies considered that there is not a clinically significant change in optical quality after V4c ICL implantation based on some parameters such as HOAs, MTF cut-off frequency and Strehl ratio. Specifically, when compared to the non-hole model, it has been found to be equivalent in these parameters, concluding that both models provide good optical quality, and that the presence of the central hole does not significantly affect the optical quality after the surgery. When compared to other corneal refractive surgical procedures, it is found that ICL implantation induced fewer HOAs than wavefront-guided LASIK or SMILE surgeries and the MTF cut-off frequency was higher than that found in SMILE. Differences between procedures, some introducing a lens in the posterior chamber (corneal incision, lens optics and position), while others modifying the corneal profile (laser ablation and flap creation) are sources of the changes reported in the optical quality.

## Data Availability

Not applicable.

## Abbreviations

ICL: Implantable collamer lens
SE: Spherical equivalent
HOAs: Higher-order aberrations
MTF: Modulation transfer function
SMILE: Small incision lenticule extraction
